# Supplementary material for: Outcomes of critically ill end-stage kidney disease patients who underwent major surgery
Source: PeerJ. 2021 May 3;9:e11324. doi: 10.7717/peerj.11324 (PMC8101474; doi:10.7717/peerj.11324)
Supplement: Supplemental Information 2 [file peerj-09-11324-s002.docx]

Databook

|  | Variable_name | Description | Code/unit |
| --- | --- | --- | --- |
| 1 | age | age | years |
| 2 | sex | sex | 1=male, 2=female |
| 3 | eskd | presence of end stage kidney disease | 1=yes, 0=no |
| 4 | bw | body weight | kg |
| 5 | ht | height | cm |
| 6 | bmi | body mass index | kg/sq-m |
| 7 | u_dm | pre-existing diabetes mellitus | 1=yes, 0=no |
| 8 | u_ht | pre-existing hypertension | 1=yes, 0=no |
| 9 | u_dlp | pre-existing dyslipidemia | 1=yes, 0=no |
| 10 | u_cad | pre-existing cardiovascular disease | 1=yes, 0=no |
| 11 | u_cva | pre-existing cerebrovascular accident | 1=yes, 0=no |
| 12 | u_ckd | pre-existing chronic kidney disease | 1=yes, 0=no |
| 13 | hos_day | hospiral length of stay | days |
| 14 | icu_day | ICU length of stay | days |
| 15 | op_time | operative time | hours |
| 16 | cr | sereum creatinine at ICU admission | mg/dL |
| 17 | egfr | estimate glomerular filtration rate | ml/min |
| 18 | icu_death | Death at ICU admission | 1=yes, 0=no |
| 19 | hos_death | Death during hospital admission | 1=yes, 0=no |
| 20 | srests | SOFA-respiratory score | score |
| 21 | slivers | SOFA-liver score | score |
| 22 | scvsbps | SOFA-cardiovascular score | score |
| 23 | ssgcs | SOFA-GCS score | score |
| 24 | splt | platelet | per cubic ml |
| 25 | splts | SOFA-coagulopathy score | score |
| 26 | scrs | SOFA-Renal score | score |
| 27 | stotals | SOFA total score | score |
| 28 | snonrenals | SOFA non-renal score | score |
| 29 | asa | ASA physical status | score |
| 30 | emer_n | Emergency surgery | 1=yes, 0=no |
| 31 | anesthesia | anesthesia technique | 1=GA, 2= RA |
| 32 | systbp | systolic blood pressure at ICU | mmHg |
| 33 | diasbp | diastolic blood pressure at ICU | mmHg |
| 34 | vasopressor | vasopressor at ICU | 1=yes, 0=no |
| 35 | type_new | type of surgery | 1=GI sx, 2= vascular sx, 3=uro sx, 5=hepato sx, 6=neuro sx, 7=CVT, 9=others |
| 36 | type1 | gastrointestinal surgery | 1=yes, 0=no |
| 37 | type2 | vascular sugery | 1=yes, 0=no |
| 38 | type3 | urological surgery | 1=yes, 0=no |
| 39 | type5 | hepato-pancreato-biliary surgery | 1=yes, 0=no |
| 40 | type6 | neurosurgery | 1=yes, 0=no |
| 41 | type7 | cardiothoracic surgery | 1=yes, 0=no |
| 42 | type9 | others surgery | 1=yes, 0=no |
| 43 | icu_day_c | ICU length of stay-censor at 30 days | days |
| 44 | c_elytes | ICU co-morbidity with electrolytes imbalance | 1=yes, 0=no |
| 45 | c_aki | ICU co-morbidity with Acute kidney injury | 1=yes, 0=no |
| 46 | c_sepsis_all | ICU co-morbidity with sepsis from every sites | 1=yes, 0=no |
| 47 | c_sepsis_pne | ICU co-morbidity with pneumonia causing sepsis | 1=yes, 0=no |
| 48 | c_uti | ICU co-morbidity with urinary tract infection causing sepsis | 1=yes, 0=no |
| 49 | c_ssi | ICU co-morbidity with soft tissue and skin infection causing sepsis | 1=yes, 0=no |
| 50 | c_sepsis_other | ICU co-morbidity with sepsis by other causes | 1=yes, 0=no |
| 51 | c_cvd_all | ICU co-morbidity with cardiovascular diseases | 1=yes, 0=no |
| 52 | c_ami | ICU co-morbidity with acute coronary syndrome | 1=yes, 0=no |
| 53 | c_chf | ICU co-morbidity with congestive heart failure | 1=yes, 0=no |
| 54 | c_arrhytm | ICU co-morbidity with cardiac arrhythmia | 1=yes, 0=no |
| 55 | c_gib | ICU co-morbidity with gastrointestinal bleeding | 1=yes, 0=no |
| 56 | c_cva | ICU co-morbidity with cerebrovascular accident | 1=yes, 0=no |
| 57 | c_dvt | ICU co-morbidity with deep venous thrombosis | 1=yes, 0=no |
